# T_1_ ‐weighted fMRI in mouse visual cortex using an iron oxide nanoparticle contrast agent and UTE imaging at 9.4 T

**DOI:** 10.1002/mrm.70103

**Published:** 2025-09-29

**Authors:** Naman Jain, Saskia Bollmann, Kai‐Hsiang Chuang, Jonathan R. Polimeni, Markus Barth

**Affiliations:** ^1^ Queensland Brain Institute The University of Queensland St Lucia Queensland Australia; ^2^ Centre for Advanced Imaging The University of Queensland St Lucia Queensland Australia; ^3^ School of Electrical Engineering and Computer Science The University of Queensland St Lucia Queensland Australia; ^4^ Athinoula A. Martinos Center for Biomedical Imaging Massachusetts General Hospital Charlestown Massachusetts USA; ^5^ Department of Radiology Harvard Medical School Boston Massachusetts USA; ^6^ Harvard‐MIT Program in Health Sciences and Technology, Massachusetts Institute of Technology Cambridge Massachusetts USA; ^7^ ARC Centre for Innovation in Biomedical Imaging and Technology The University of Queensland St Lucia Queensland Australia; ^8^ Present address: Max Planck Institute for Biological Cybernetics Tübingen Germany; ^9^ Present address: Richard M. Lucas Center for Imaging, Department of Radiology Stanford University School of Medicine Stanford California USA

**Keywords:** bright‐blood signal, CBV‐fMRI, contrast agent, functional hyperemia, high‐resolution fMRI, non‐BOLD fMRI

## Abstract

**Purpose:**

This study aims to investigate the feasibility of using T_1_‐weighted fMRI with an iron oxide nanoparticle contrast agent and UTE imaging at 9.4 T to measure functional hyperemia in the mouse visual cortex. The goal is to capture positive signal changes in both the parenchyma and pial surface, and to test whether surface vessels respond during neuronal activation.

**Method:**

The study involved scanning of nine mice after administration of iron oxide–based superparamagnetic contrast agent via the tail vein. Two functional imaging experiments were conducted: one to investigate the effect of TE on the functional response, and another to characterize the impact of higher resolution on UTE functional contrast. Regions of interest were defined in the parenchyma and pial surface of the visual cortex.

**Results:**

The administration of the contrast agent produced a bright‐blood signal in the vasculature in structural MRI when using a UTE acquisition. Positive signal changes were observed at the shortest TE (0.164 ms) in voxels sampling both the parenchyma (0.22% ± 0.08%) and pial surface (0.26% ± 0.1%), providing evidence that UTE fMRI experiments can detect changes in both parenchymal and pial vessels. Measurements using longer TEs (≥1 ms) showed negative signal changes, as expected. Higher spatial resolution resulted in increased percent signal change at the pial surface, suggesting reduced partial volume effects.

**Conclusion:**

The findings demonstrate that T_1_‐weighted fMRI with UTE imaging and Superparamagnetic Iron Oxide nanoparticles captures positive signal changes across all vascular compartments, providing additional insights into the involvement of surface vessels during functional hyperemia.

## 1 INTRODUCTION

The roles that different vascular compartments play during functional hyperemia have been debated extensively in the field of functional neuroimaging.^1^, ^2^, ^3^, ^4^, ^5^, ^6^ In vivo measurements of various hemodynamic components can aid in addressing the question of how these compartments are engaged and interact during functional hyperemia, and thereby provide deeper insight into the vascular response to neuronal activity and improve the interpretation of functional MRI signals. Changes in cerebral blood volume (CBV) within arteries and small arterioles are of particular interest^5^, ^7^ because these constitute the “active” component of the hemodynamic response from which changes in cerebral blood flow and oxygenation follow.^8^, ^9^, ^10^, ^11^, ^12^, ^13^

MRI‐based CBV measurements in small‐animal models typically use iron oxide–based contrast agents injected into the bloodstream.^14^, ^15^, ^16^, ^17^ The paramagnetic properties of iron oxide nanoparticles^18^, ^19^, ^20^ induce a dephasing of spins in and around blood vessels in gradient echo–based acquisitions, creating a black‐blood contrast and thus generating a signal decrease proportional to the increase in CBV^21^ during functional hyperemia. CBV‐fMRI has been shown to provide a higher contrast‐to‐noise ratio than conventional BOLD‐fMRI^22^ and, because of the strong signal dephasing around large pial vessels, signal changes at the cortical surface are suppressed resulting in higher specificity to the parenchymal microvasculature.^21^ CBV‐fMRI—with or without contrast agent—has been used to study brain function^23^, ^24^, ^25^, ^26^, ^27^ and showed improved spatial localisation^21^, ^23^, ^24^, ^28^ due to the apparent insensitivity to midsized and large vessels, especially the large draining veins on the pial surface of the cortex that dominate the BOLD contrast.^21^

Optical imaging techniques such as two‐photon microscopy also allow the observation of vascular responses to functional hyperemia.^29^ Beyond changes in oxygenation, direct measurements of changes in vascular diameter of individual vessels, which are related to changes in vascular volume and thus CBV, can be made.^10^ Two optical imaging studies^29^, ^30^ reported large diameter changes mostly in surface arteries, showing a discrepancy between CBV changes observed with optical imaging and those observed using iron oxide nanoparticles as a contrast agent in fMRI. Note, however, that signal changes at the pial surface have been occasionally observed in CBV‐fMRI studies as well.^23^, ^24^, ^27^, ^31^, ^32^

In addition to their well‐known $T_{2}^{*}$ shortening effect, iron oxide nanoparticles also cause a marked T_1_ shortening.^28^, ^33^ This can be exploited to create a bright‐blood contrast when sufficiently reducing dephasing ($T_{2}^{*}$) effects by minimizing the TE, for example, via zero TE (ZTE)^34^ or UTE sequences.^33^, ^35^, ^36^ Examples of structural vascular imaging were shown by Gharagouzloo et al.^35^, ^36^ using a 3D UTE sequence. In a recent conference proceeding, one group has demonstrated the potential of combining an iron oxide–based superparamagnetic contrast agent and ZTE for fMRI showing positive signal changes in stimulated brain areas in rats.^34^

Motived by this, the goal of our study was to further investigate T_1_‐weighted fMRI responses using an iron oxide–based superparamagnetic contrast agent and a short‐TE acquisition. To achieve a high in‐plane resolution while maintaining reasonably short TR, we chose a 2D UTE acquisition scheme. To compare our findings with those of previous studies^33^, ^37^, ^38^, ^39^ and to better understand the transition from a T_1_‐weighted image contrast at short TEs to a $T_{2}^{*}$‐weighted image contrast at later TEs, we repeated the functional experiments across a range of TEs (from 0.164 to 10 ms). Whereas in predominantly T_1_‐weighted images the blood vessels are expected to exhibit a bright signal, they are expected to appear dark in the predominantly $T_{2}^{*}$‐weighted images as used in classical CBV imaging. Notably, CBV changes in large vessels should be preserved UTE because little‐to‐no dephasing occurs in the predominantly T_1_‐weighted images. To better understand the contributions of different vascular compartments in each of these conditions, we analyzed the task response in the parenchyma as well as the pial surface of the visual cortex, assuming the parenchyma mainly contains capillaries as well as small arterioles and venules, and the pial surface predominantly larger vessels. Lastly, we also explored the possibility of even higher spatial resolution to reduce partial volume effects on the pial surface by performing the experiment at the shortest achievable TE with an in‐plane resolution of 0.1 mm.

## 2 METHODS

### 2.1 Animals and ethics

All animal experiments were conducted with the approval of the local animal ethics committee. Imaging data from nine male C57BL/6J (age: 8 to 10 weeks) mice were acquired.

For preparation, the animals were anesthetized with 3% isoflurane mixed with air and oxygen in a 2:1 ratio until unconscious. The iron oxide–based superparamagnetic contrast agent Molday ION (BioPAL, Worcester, MA, USA; 5 mg Fe/ml) was administered into the tail vein by a senior animal technician with a dose of approximately 29.5 mg/kg. For sedation, a medetomidine (Troy Laboratories, Glendenning, NSW, Australia) bolus of 0.03 to 0.05 mg/kg was injected intraperitoneally.

For imaging, mice were positioned on an animal cradle, and their heads were fixed using a bite bar and ear bars to limit movement. Vital parameters such as respiration rate, body temperature, heart rate, and blood oxygenation levels were continuously monitored by a veterinary support officer throughout the imaging session. A respiration sensor was placed beneath the belly of the mice, a rectal temperature probe was used to monitor body temperature, and a tail‐cuff SpO_2_ sensor was employed to measure both heart rate and blood oxygenation level (model 1030, SAII, Stony Brook, NY, USA). Body temperature was maintained at 36 to 37°C by using a warm‐water cushion for which the temperature could be adjusted based on the body temperature of the mice. Respiration rate was maintained at 120 to 150 breaths per min, heart rate at 210 to 230 beats per min, and SpO_2_ levels at >96% by adjusting the isoflurane concentration between 0.2% and 0.5%. Medetomidine was supplied constantly during imaging through an intraperitoneal infusion at 0.085 to 0.1 mg/kg/h 10 min after the bolus using an infusion pump.

### 2.2 MRI data acquisition

Imaging data were acquired on a 9.4 T preclinical MRI system (Biospec 9.4/30, Bruker BioSpin GmbH, Ettlingen, Germany) controlled by a Avance III console running Paravision 6.0.1. Maximum gradient strength and slew rate were 660 mT/m and 6000 mT/m/s, respectively. A Bruker desinged and built 86‐mm quadrature volume coil was utilized for transmission, and an in‐house–developed 10‐mm ellipsoid surface coil was used for reception.

An anatomical reference image was acquired using a T_2_‐weighted TurboRARE (rapid acquisition with relaxation enhancement) sequence with a TE of 55 ms, a voxel size of 0.1 × 0.1 × 0.25 mm^3^, 24 slices, and three averages with a total acquisition time of 6 min and 36 s. A map of the vasculature was obtained in five mice using a 3D UTE sequence with a TE of 13 μs, a TR of 5 ms, an isotropic nominal resolution of 0.1 mm, 115,432 radial spokes, a final matrix size of 192 × 192 × 192, and a total acquisition time of 9 min and 37 s.

Two different functional imaging experiments were performed, with animals divided into two groups corresponding to the two experiments with different spatial resolutions: Group 1–high resolution used 0.2 × 0.2 × 0.5 mm^3^ (experiment 1); and group 2–ultrahigh resolution used 0.1 × 0.1 × 0.5 mm^3^ (experiment 2). In experiment 1, the effect of TE on the functional response was investigated in the five mice of group 1 by acquiring functional data with a voxel size of 0.2 × 0.2 × 0.5 mm^3^ at five different TEs using either a 2D UTE (0.164 ms, 0.5 ms, 1 ms) or a 2D FLASH (4 ms and 10 ms) sequence (see Table 1). To minimize $T_{2}^{*}$ contamination in the UTE acquisitions, the shortest possible readout durations of 0.32 ms for the high‐resolution acquisition and 0.96 ms for ultrahigh‐resolution acquisition were used.

**TABLE 1:** Comparison of protocol parameter values between UTE and FLASH acquisitions.

	2D UTE (ultrahigh resolution)	2D UTE (high resolution)	2D FLASH
No. of animals	**4**	**5**	**5**
TE	0.164 ms	0.164 ms, 0.5 ms and 1 ms	4 ms and 10 ms
TR	4.968 ms	4.968 ms	55.556 ms
Volume TR	3 s	3 s	3 s
Number of slices	1	2	8 (TE = 4 ms) or 4 (TE = 10 ms)
Resolution	0.1 × 0.1 × 0.5 mm^3^	0.2 × 0.2 × 0.5 mm^3^	0.2 × 0.2 × 0.5 mm^3^
Slice gap	NA	0.1 mm	0.1 mm
Matrix size	192 × 192	96 × 96	64 × 54
Number of radial spokes	604	302	NA
Readout bandwidth	100 000 Hz	100 000 Hz	50 000 Hz
Readout duration	0.96 ms	0.32 ms	1.28 ms

To ensure a sufficiently short‐volume TR of 3 s compatible with the timing of the hemodynamic response, the coverage of the 2D UTE acquisition was limited to two slices, and the number of slices in the FLASH acquisitions was set to either eight (TE = 4 ms) or four (TE = 10 ms) to match the volume TR. A system‐calculated RF excitation pulse with a flip angle (FA) of 15° was used to acquire the FLASH images; and to achieve shortest TE for the UTE images, a half‐Gauss RF excitation pulse with an FA of 20° was used with the FA optimization based on previous work.^35^ The FLASH acquisition with TE = 4 ms was performed first to serve as a functional localizer, and the two slices with the strongest responses were chosen to plan all subsequent functional scans. The order of the remaining scans was pseudo‐randomly alternated for each mouse. Before each 2D UTE acquisition, an online k‐space trajectory adjustment with 32 averages was performed. As a control, one additional mouse was scanned without contrast injection.

In experiment 2, the effect of higher spatial resolution was investigated in the four animals of group 2. First, the FLASH sequence with 4 ms TE was used as a functional localizer. Then, single‐slice 2D UTE images with the shortest TE of 0.164 ms and a voxel size of 0.1 × 0.1 × 0.5 mm^3^ were acquired. To compensate for the reduction in SNR due to the smaller voxel volume, we acquired more runs to provide noise cancellation through data averaging (four mice in total; with two runs in two mice and four runs in two mice).

### 2.3 Stimulation paradigm

The visual stimulus consisted of a blue flashing light delivered through an optical fiber (frequency 5 Hz, pulse duration 10 ms, power <1 mW), which was previously shown to elicit robust positive BOLD response.^40^ To minimize the risk of eye damage or discomfort, the optical fiber was pointed toward the rear of the scanner bore to avoid projecting light directly into the eyes of the animals. The stimulation paradigm utilized a block design, with eight blocks of 30‐s rest interspersed with seven blocks of 30‐s stimulation. In total, each run lasted 7 min and 30 s, and a 5‐min rest period was inserted between runs. In experiment 1, one run was acquired per TE and animal; in experiment 2, one run for the functional localizer and between two to four runs per animal were acquired for the shortest TE (0.164 ms).

### 2.4 Activation map estimation

FMRIB Software Library (FSL)^41^, ^42^ (https://fsl.fmrib.ox.ac.uk/fsl) and Analysis of Functional Neuroimages (AFNI)^43^, ^44^ (https://afni.nimh.nih.gov/) software packages were utilized for data processing. The first three volumes of each run were discarded to ensure the MR signal had reached steady state. Motion correction was applied using *3dvolreg* from the AFNI package, and Fourier interpolation was used for resampling. The middle volume was selected as the reference image for motion correction. To enhance the SNR for extracting activation maps, spatial smoothing with a FWHM equivalent to two voxels was applied, that is, 0.4 mm for the high‐resolution data and 0.2 mm for the ultrahigh‐resolution data.

### 2.5 ROI definition and analysis

Percent signal changes of the fMRI data were investigated in two regions of interest (ROIs), namely the parenchyma and the pial surface of visual cortex. The ROIs were manually defined on the TurboRARE anatomical reference image guided by the Paxinos and Franklin mouse brain atlas. The parenchymal ROI was placed centrally in cortical gray matter, leaving a two‐voxel‐wide gap between this ROI and the cortical surface. A one‐voxel‐thick pial surface ROI was then placed above the parenchymal ROI. On average, 21 voxels (in native fMRI space) contributed to the parenchymal ROI and nine voxels (in native fMRI space) contributed to the pial surface ROI.

To minimize the need for manual realignment, similar image contrasts within each sequence type, UTE (TE: 0.164, 0.5 and 1 ms) or FLASH (TE: 4 and 10 ms), were first concatenated and then motion corrected. This resulted in one mean functional image per sequence type, which was then manually aligned to the anatomical reference image using ITK‐Snap,^45^ producing a rigid transformation matrix. The AFNI *3dresample* function was then used to resample the functional time series into the space of the anatomical reference image. To minimize bias when aligning the mean functional and structural images due to the signal decay at the surface of the images with longer TE, the skin and fat signal of the skull were used as landmarks.

To estimate the trial responses within the ROIs for each individual TE value, the demeaned time series across the seven trials in each run and within each ROI were averaged. Percent signal change was then computed by subtracting and then dividing by the averaged signal time course by the baseline signal level (approximated as the mean intensity of the last 6 data points immediately before stimulus onset in all trials for each run). From this, the average percent signal change was computed by averaging across 35 time points acquired during the stimulus presentation.

For comparison, we also computed the absolute image intensity in the pial surface ROI relative to the image intensity within the parenchyma of the visual cortex by scaling the average image intensity values in the parenchymal ROI to 1 and then applying the same scaling factor to the pial surface ROI. This enabled a comparison of differences in percent signal change relative to differences in image intensity.

### 2.6 Analysis of the ultrahigh‐resolution UTE data

The analysis of the UTE data acquired with 0.1‐mm in‐plane resolution followed a similar approach as outlined above. Because only a single 2D image slice was acquired, *3dAllineate* was used for motion correction because it supports alignment of single 2D images. The same procedure as for the high‐resolution data was employed to compute the trial responses for both ROIs.

### 2.7 Statistical analysis

To analyze the task fMRI time series data, a design matrix was created that included a boxcar waveform. We utilized a hemodynamic response function tailored to the mouse cortex; the hemodynamic response function was modeled as a gamma‐variate function with a phase of 0 s, SD of 2 s, and mean lag of 4 s. Temporal derivatives were added to the model, and a high‐pass filter with a cutoff of 90 s was applied. Data were analyzed using a general linear model (GLM), as implemented in the GLM setup package in FSL. First‐level GLM analysis was performed for each subject through GLM setup package in FSL. This was done to generate z‐maps for individual subjects. z‐maps were plotted in the FSLeyes, and a z‐value of 1.6 (corresponding to uncorrected *p* < 0.05) was regarded as statistically significant.

## 3 RESULTS

**FIGURE 1:**
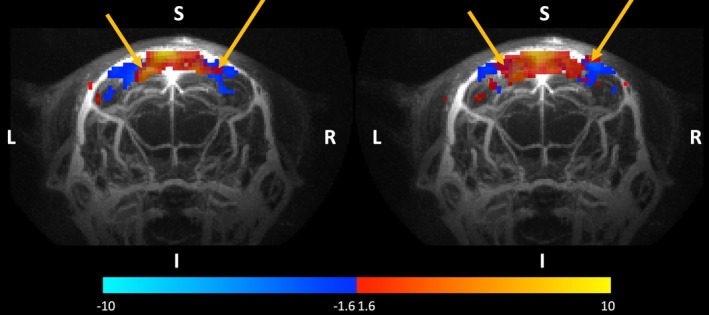
Activation maps (*z*‐values thresholded at *p* < 0.05) from one animal in group 1 showing responses to visual stimulation across longest and shortest TEs acquired with a 2D UTE sequence (0.164 ms TE) or a 2D FLASH sequence (10 ms TE) after contrast agent administration with an underlay showing maximum‐intensity projection of a whole mouse brain acquired using 3D UTE sequence after iron oxide base contrast agent injection. Yellow arrows indicate overlapping responses in visual cortex using either TE.

The administration of the iron oxide–based superparamagnetic contrast agent reduced the T_1_ of blood and produced a bright‐blood signal in the vasculature when using the UTE acquisition, as previously shown.^35^ Figure 1 illustrates this effect, showing a whole‐brain maximum‐intensity projection of a 3D UTE image, acquired with a TE of 13 μs, from one mouse. Figure 1 shows high spatial overlap between large vessels and the observed activation maps at the shortest TE, indicating indeed the ability of UTE imaging with contrast agent to resolve larger macrovessels.

**FIGURE 2:**
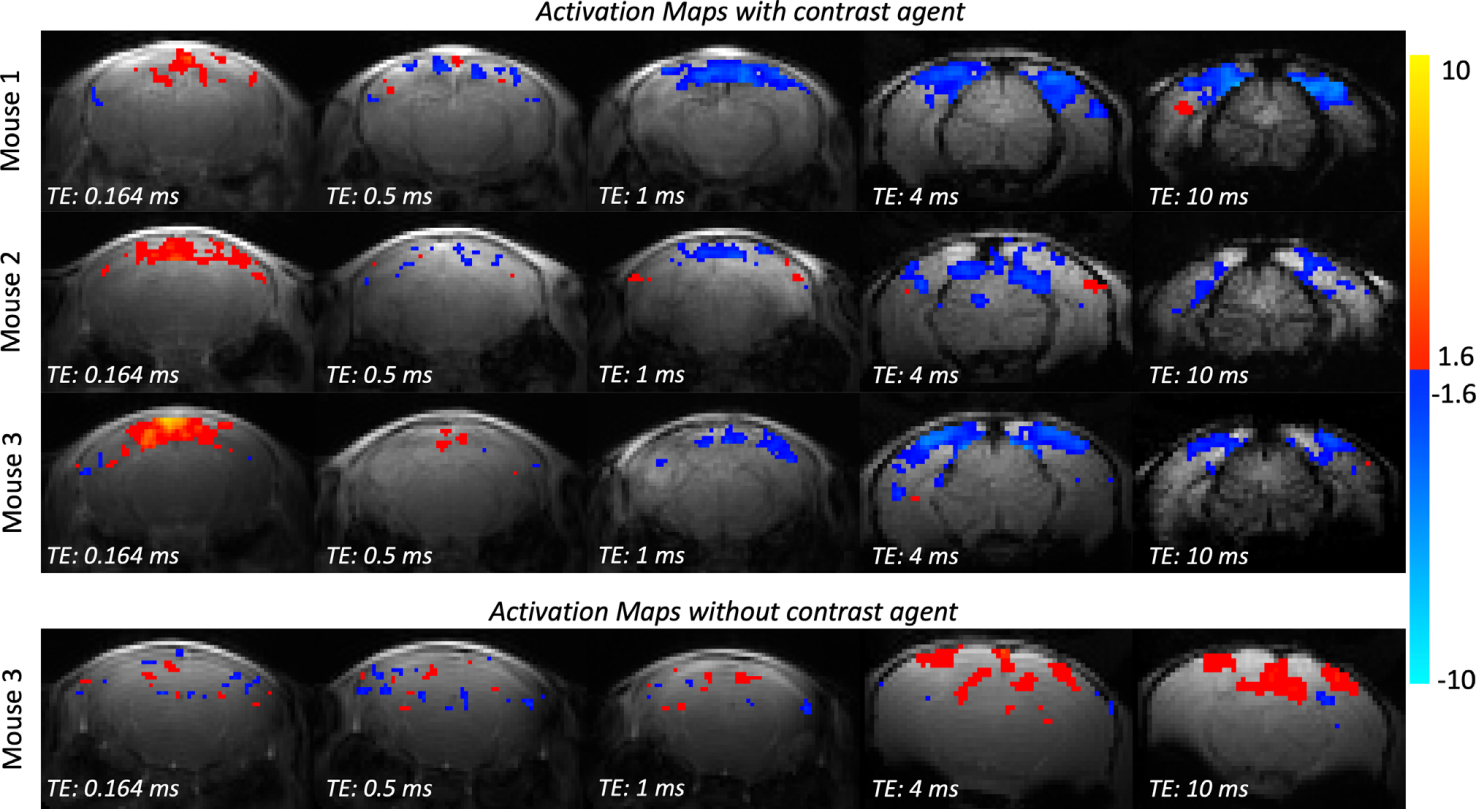
Activation maps (*z*‐values thresholded at *p* < 0.05) from three animals in group 1 showing responses to visual stimulation across five different TEs acquired with a 2D UTE sequence (0.164, 0.5, and 1 ms TE) or a 2D FLASH sequence (4 and 10 ms TE) after contrast agent administration (rows 1–3). The data acquired with the shortest TE exhibit positive signal changes (positive *z*‐values overlayed in hot color scale), whereas data using longer TEs exhibit negative signal changes (negative *z*‐values overlayed in cold color scale). The bottommost row shows activation maps (*p* < 0.05) for the control experiment without contrast agent. For data acquired with short TEs (≤1 ms), no distinct activation was found, whereas in data acquired at longer TEs, positive signal changes in visual cortex were observed consistent with a BOLD response.

**FIGURE 3:**
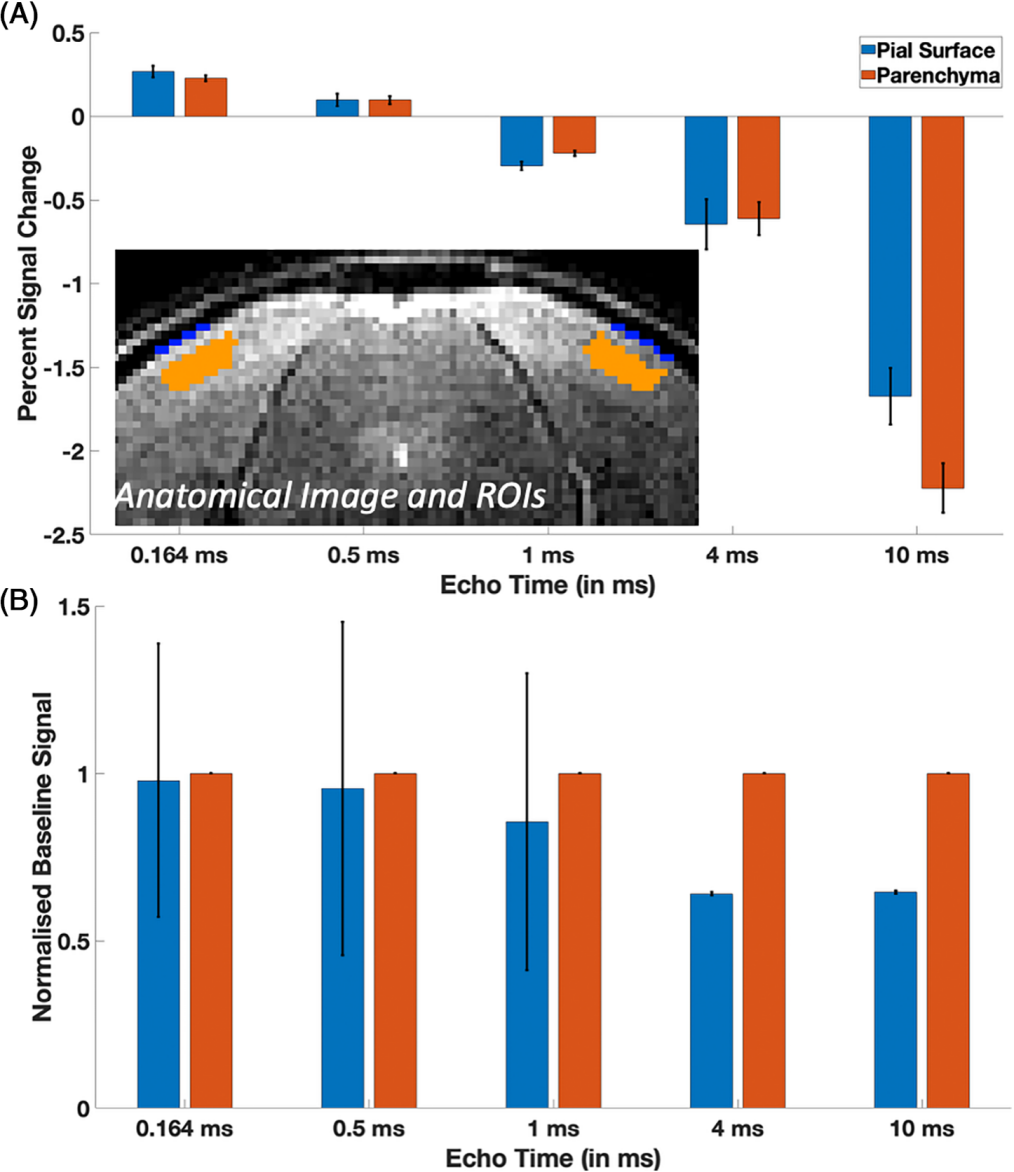
(A) Percent signal change (*n* = 5) as a function of TE after contrast injection measured with a 2D UTE (TE ≤1 ms) or 2D FLASH (TE >1 ms) sequence. At short TE values, positive percent signal changes were small but of similar magnitude on the pial surface (0.26% ± 0.03%) and in the parenchyma (0.22% ± 0.01%). With increasing TE, percent signal change decreased and reached strong negative values of similar magnitude (pial surface: −1.67% ± 0.16% and parenchyma: −2.22% ± 0.14%) at the longer TEs (TE >1 ms) in both the parenchyma and on the surface. Error bars indicate standard error of the mean across five subjects. (B) Normalized image intensities averaged within both ROIs, scaled such that values in the parenchymal ROI always equal 1, show a steady decrease in the pial surface ROI with TE value. Error bars again indicate standard error of the mean across five subjects. (Inset: Pial and parenchymal ROIs marked on the anatomical image). ROI, region of interest.

When comparing the functional responses to visual stimulation across five different TEs ranging from 0.164 to 10 ms, positive signal changes were found in all mice at the shortest TE, and negative signal changes were found at longer TEs (TE ≥1 ms) (Figures 2 and 3A). The data acquired with the two longest TEs exhibit the expected negative signal change with activation^21^, ^25^, ^28^ in mouse visual cortex, whereas the activation region observed in the data acquired at TE = 1 ms included visual cortex but also the superior sagittal sinus. No clear activation pattern was seen in data acquired at the intermediate TE of 0.5 ms. As a control, we also repeated the experiment without contrast agent in one subject and acquired data using the same acquisition protocols. There, positive BOLD activation patterns in visual cortex were present for the two longest TE values, but no consistent responses were found at shorter TEs (TE ≤1 ms).

To investigate the effect of TE on the response strength within the parenchyma of the visual cortex and at its pial surface, percent signal change across the five TE values for each ROI were compared (Figure 3A). In the data acquired with the shortest TE, small positive signal changes of similar magnitude in both ROIs were observed (pial surface [0.26% ± 0.03%] and in the parenchyma [0.22% ± 0.01%]), averaged across the five mice of group 1). Within both the pial surface and parenchyma ROIs, the percent signal change consistently decreased with TE and reached a negative value of about −1.7% at the longest TE. Figure 3B shows a steady decrease in image intensity of the pial surface ROI relative to the parenchymal ROI with increasing TE values.

**FIGURE 4:**
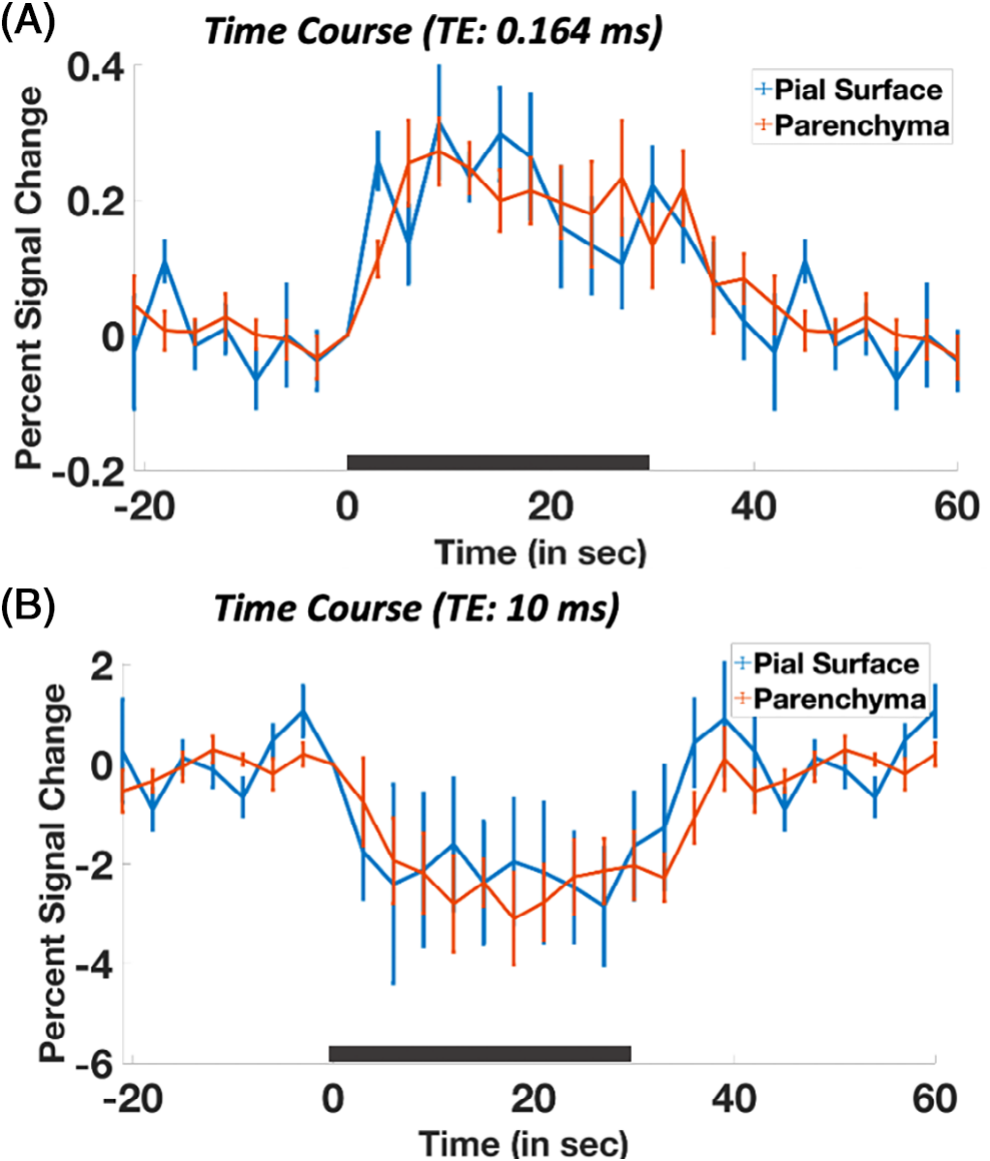
Trial‐averaged time courses (n = 5) within the pial surface ROI (blue) and parenchymal ROI (orange) estimated from data acquired at the shortest (A) and longest (B) TEs. Error bars indicate standard error of mean across five subjects. Black bar indicates stimulus ON period.

The trial‐averaged fMRI responses are presented in Figure 4 both for the predominantly T_1_‐weighted data (Figure 4A) corresponding to the UTE acquisition with the shortest TE value, and for the predominantly $T_{2}^{*}$‐weighted data (Figure 4B) corresponding to the FLASH acquisition with the longest TE value. Responses are plotted for both the parenchyma and pial surface ROIs. As seen in Figure 3, these responses show a positive signal change in the T_1_‐weighted data and a negative signal change in the $T_{2}^{*}$‐weighted data. Both the amplitudes and timings of the responses seen in the parenchymal and pial surface ROIs are comparable within each acquisition, although subtle differences in timing may be appreciated between the responses measured with the T1‐weighted data and the $T_{2}^{*}$‐weighted data. T1‐weighted fMRI exhibits slower and delayed temporal responses compared to BOLD fMRI owing to the longer longitudinal relaxation time constants. This reduced temporal resolution can attenuate and delay hemodynamic events.

**FIGURE 5:**
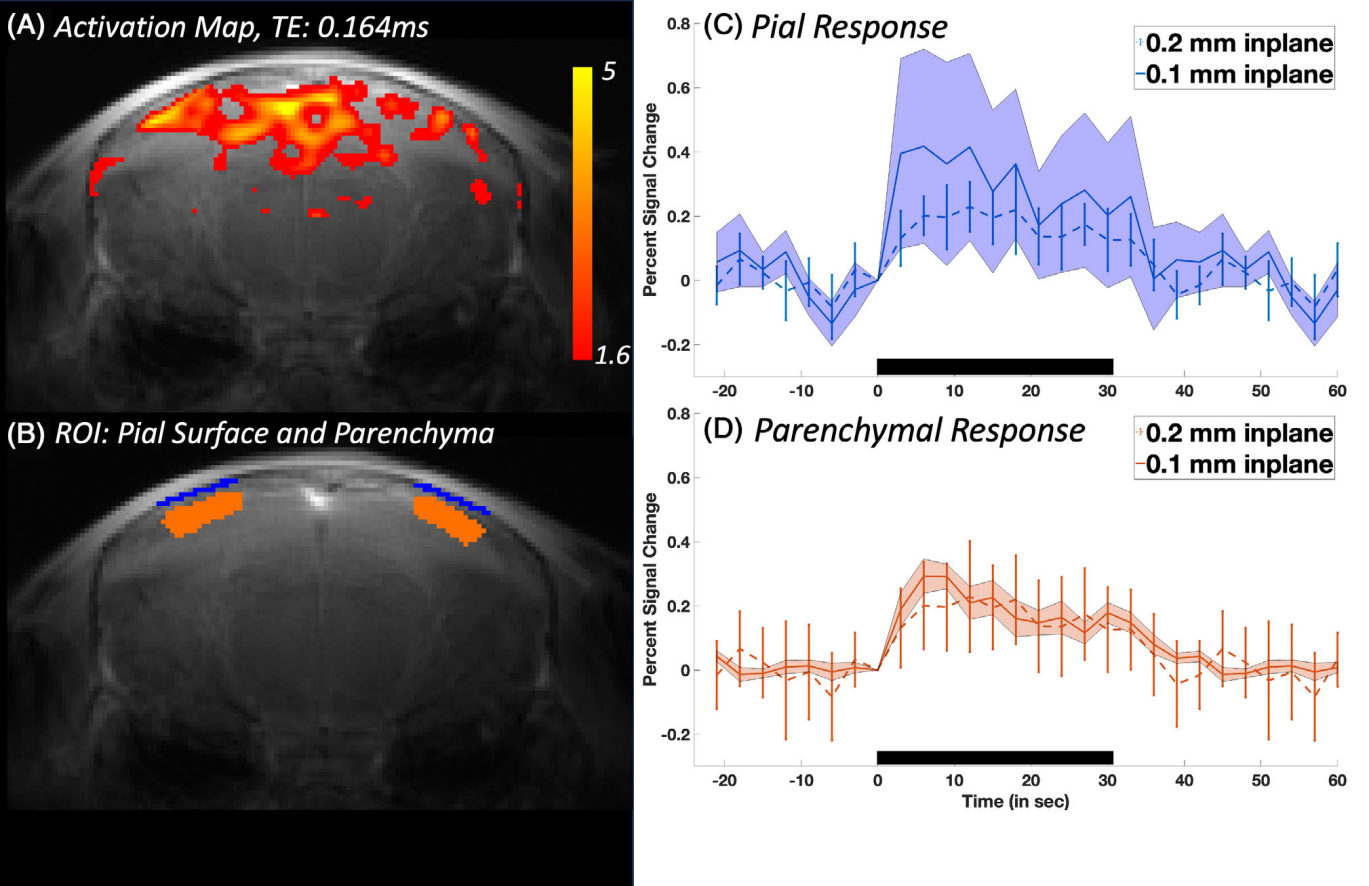
Comparison of UTE fMRI responses between the high‐resolution and ultrahigh‐resolution fMRI acquisitions. (A) Example activation maps in one subject at ultrahigh resolution. (B) ROIs at pial surface (blue) and parenchyma (orange) overlaid on the mean of the ultrahigh‐resolution fMRI data. (C and D) Comparison of average time courses extracted from the pial surface (C) and parenchyma (D) ROIs using UTE, TE = 0.164 ms, for both the high‐ (dashed lines) and ultrahigh‐resolution (solid lines) fMRI data (groups 1 and 2). The trial responses are plotted as percent signal change averaged across all subjects (*n* = 5 in group 1, *n* = 4 in group 2) for the pial surface (mean PSC: 0.47% ± 0.11%) and parenchyma (mean PSC: 0.21% ± 0.02%) ROIs across both spatial resolutions: 0.2‐mm resolution (dashed line) and 0.1‐mm resolution (solid line). Black bar indicates stimulus duration; error bars and shading both represent standard error of the mean across subjects. PSC, percent signal change.

Figure 5A shows an example activation map of a UTE acquisition at ultrahigh resolution (0.1‐mm) depicting widespread significant functional activation across the cortex (Figure 5A); for reference, Figure 5B shows the two ROIs overlaid on ultrahigh‐resolution UTE‐fMRI data. Figure 5C,D show the trial responses for both the high‐ and ultrahigh‐resolution fMRI data (plotted on the same axes for comparison) from both the parenchyma and pial surface ROIs. In data averaged within the parenchymal ROI, we observed a similar response amplitude and shape for both imaging resolutions (Figure 5D), whereas in data averaged within the pial surface ROI, the mean response amplitude of the ultrahigh‐resolution acquisitions (0.1 mm) was about twice as high as the response compared to the high‐resolution acquisitions (0.2 mm) but also more variable across mice (see Section 4).

## 4 DISCUSSION

In this study, we investigated the characteristics of fMRI signal changes after injection of the iron oxide–based superparamagnetic contrast agent Molday (BioPAL) ION using a UTE acquisition scheme. This combination of Molday ION and a UTE acquisition has been previously used in vascular anatomical imaging,^35^ where it was shown to generate a bright‐blood signal due to the T_1_‐shortening property of MION when injected into the blood stream (as also shown in Figure 1). This is in distinction to “classical” iron oxide nanoparticle experiments^22^, ^25^, ^28^, ^33^, ^39^, ^46^ that use the $T_{2}^{*}$‐shortening properties of the contrast agent in combination with $T_{2}^{*}$‐weighted MRI acquisitions.

### 4.1 Effect of TEs on contrast mechanisms

When varying the TE from 0.164 to 10 ms, we observed a reversal in functional contrast from positive (i.e., T1‐weighted) to negative (i.e., $T_{2}^{*}$‐weighted) signal change at around 0.5 to 1 ms (Figure 2). The functional response in the $T_{2}^{*}$‐weighted images was fairly well localized to the visual cortex (4 and 10 ms in Figure 2), as expected,^16^, ^17^, ^47^ whereas the response in the T1‐weighted image (0.164 ms in Figure 2) was more spread out and also less consistent across mice. This could be due to the reduction in image intensity on the cortical surface relative to parenchyma (Figure 3B), presumably due to the dephasing effects around large vessels, also known as *vascular filter*.^21^ Our results show that the percent signal change at the pial surface and within the parenchyma at shortest TE was similar (Figure 3A). Further, the magnitude of the $T_{2}^{*}$‐weighted percent signal change was higher than the T1‐weighted signal change (Figure 3), indicating higher sensitivity and potentially lower noise variance, partly explaining the higher consistency. Further, we observed a lower percent signal change on the pial surface compared to the parenchyma at the longest TE, which could originate from additional positive BOLD signal contributions offsetting the contrast agent–induced CBV‐weighted signal decrease.^48^

Also, the variability observed in the surface ROI for UTE is visible in baseline signal and not in the stimulus‐evoked responses. This can be attributed to the properties (inhomogeneity, steep signal dropoff) of RF surface coils and the contrast properties of UTE imaging, where blood vessels appear as hyperintense structures.

We employed a dedicated processing pipeline to minimize variance due to ROI selection by marking only one parenchymal and one pial ROI per mouse, aligning each sequence type (UTE or FLASH) across TEs. As such, we only had to align two (instead of five) mean UTE or FLASH images manually to the structural reference image. Due to the limited coverage (two or one slice(s), respectively) and the registration between vastly different image contrasts (T_1_ to T_2_, or $T_{2}^{*}$ to T_2_), this step could only be successfully performed manually. Thus, we paid particular attention to features outside of the brain, such as fat and skull signal, to mitigate the artefactual shift in the location of the pial surface due to the signal decay in the FLASH images.

The temporal characteristics of the parenchymal and pial response at the longest and shortest TE appeared very similar (Figure 4); however, the percent signal change in the T_1_‐weighted fMRI data was much lower compared to the $T_{2}^{*}$‐weighted images. Whereas there is no comparable study for the T_1_‐weighted data, at the longer TE (TE: 10 ms), Zhao et al. observed a similar percent signal change on the pial surface and within the parenchyma in cats.^27^ Similarly, Keilholz et al. 2006^25^ reported up to 8.7% signal change in rat somatosensory cortex upon 30 mg Fe/kg injection. However, it has been shown that rat visual cortex exhibits small hemodynamic responses compared to somatosensory cortex. Further, cats utilize their visual system extensively compared to mice. Accordingly, we conclude that part of the smaller observed percent signal change is due to the different species and brain region.

Interestingly, numerous studies have reported a rapid and transient increase in arterial diameter in response to sensory stimulation, which may contribute to the observed response at the pial surface.^2^, ^12^, ^48^, ^49^, ^50^, ^51^ Notably, Drew et al.^29^ also documented changes in venous diameter with longer duration stimuli in vessels near the pial surface, which is particularly relevant given that our signal reflects total CBV, suggesting contributions from both dilating arteries and veins at the pial surface.

### 4.2 Effect of spatial resolution on signal changes

When increasing the in‐plane resolution to 0.1 mm (Figure 5), we found similar activation patterns as seen in the 0.2‐mm data (Figure 2). The estimated percent signal change within the parenchyma was also comparable between both resolutions. However, the percent signal change at the pial surface increased when reducing the voxel size. The observable difference in the response on the pial surface is consistent with a reduced partial volume effect. However, to fully resolve individual pial arteries, voxel sizes of 34 to 41 μm in mice^52^ and 50 to 300 μm in humans would be necessary. Whereas initial studies have shown the potential to resolve these anatomically,^53^ adapting this anatomical acquisition into a functional acquisition would be needed; thus, resolving these small vessels both spatially and temporally remains challenging. Accordingly, CBV‐fMRI may continue to rely on partial‐volume models for interpretation and quantification.^31^ Importantly, whereas a two‐compartment model describing gray matter and blood volume changes may be sufficient within the parenchyma, volume changes in CSF and the potential dilation of gray matter with activation also need to be considered on the surface.^54^

### 4.3 Contribution of blood flow

**FIGURE 6:**
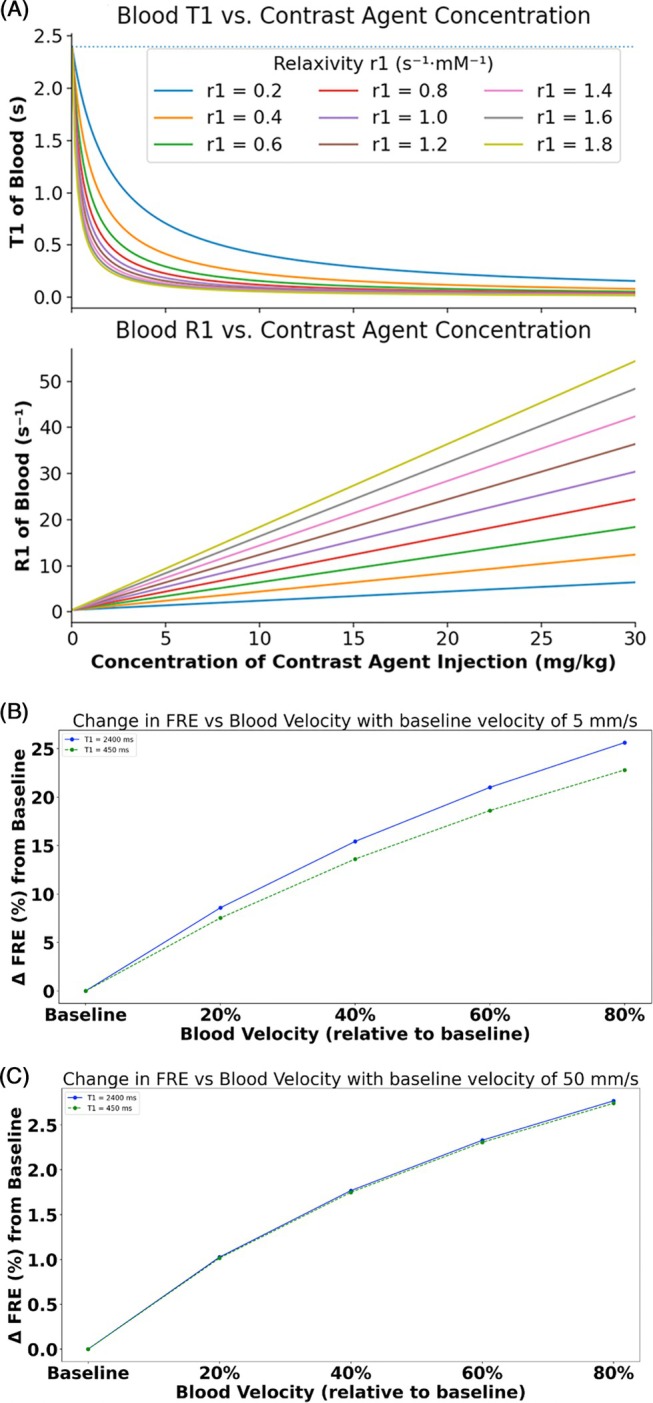
Effect of contrast agent concentration and RF pulse train length on blood T_1_ and FRE. (A) Simulated T_1_ and R_1_ relaxation time of blood as a function of contrast agent concentration for a range of baseline longitudinal relaxivity (*r*_1_) values ranging from 0.2 to 1.8 s^−1^·mM^−1^. Increasing contrast concentration reduces T_1_ nonlinearly, whereas R_1_ is increased nonlinearly, with stronger effects at higher relaxivity. Blue dashed line indicates the T_1_ of blood before injection of contrast agent. Bottom panel: Change in FRE with respect to increase in blood velocities with respect to baseline. (B) When baseline of blood was assumed to be 5 mm/s and (C) 50 mm/s. FRE, flow‐related enhancement.

In previous studies, a large 3D volume was excited to limit signal enhancements flowing into the imaging volume of interest,^55^ whereas here we only used one or two thin slice(s). Thus, the observed signal changes likely constitute a combination of both CBV changes and inflow effects. In our T_1_‐weighted data, activation would be expected to increase with CBV and inflow velocity, and so both physiological effects would contribute to an increase in signal and thus a positive response to activation. Indeed, at the shortest TE we also observed a positive response in the superior sagittal sinus. Simulations suggest 5% to 20% singal increase in slow‐flowing vessels (20% to 60% velocity increase at a baseline velocity of 5 mm/s^8^, Figure 6B) and a 1% to 2% signal increase in fast‐flowing vessels (20% to 60% velocity increase at a baseline velocity of 50 mm/s, FIgure 6C) for both conditions with and without contrast agent. This is above the percent signal increase observed here, and not in agreement with the absence of an effect in the—albeit singular—control experiment. Further, we observed nearly equal percent signal changes in the parenchyma and on the pial surface, with presumably different velocities, although this is also modulated by blood volume fraction. To mitigate this confound in future studies, further pulse sequence development is required. This could involve either significantly accelerating the acquisition of whole‐head 3D volumes to intrinsically minimize inflow‐related artifacts or implementing spatially selective saturation pulses outside the imaging volume to drive inflowing blood spins toward a steady‐state magnetization prior to entry (Figure 6).

Although we did not observe clear activation in the shortest TEs in our control data, which were acquired without a contrast agent, the T_1_ shortening induced by the contrast agent is also expected to increase inflow contrast, and so these non‐contrast control data cannot completely rule out inflow contributions in our contract‐enhanced data. Future studies could address this confound by either increasing the coverage or applying additional saturation pulses to the non‐imaged regions to reduce inflow and isolate the CBV component.

### 4.4 Involvement of different vascular compartments

Commonly, the decrease in signal with activation is interpreted as being proportional to increases in CBV.^28^ In our study, when employing longer TE (>1 ms) we were able to replicate this behavior. However, when employing an UTE using a 2D UTE sequence, we instead observed positive functional signal changes upon visual stimulation. One possible interpretation of this positive signal change is also as an increase in CBV, as suggested in the study of Gharagouzloo et al.^36^ Mapping changes in CBV using T_1_‐weighting allows for observation of contributions from large vessels that can be obscured when using $T_{2}^{*}$‐weighting (also known as *vascular filter*).^51^ This filter present in conventional $T_{2}^{*}$‐weighted contrast‐enhanced CBV‐fMRI may be beneficial for studies targeting small vessels in the parenchyma^24^, ^28^, ^56^, ^57^ to maximize neural specificity but may obscure the involvement of larger macrovessels in functional hyperemia if the aim is to understand the neuro*vascular* response. The responses at the longest TE were mostly localized to the parenchyma of the visual cortex (Figure 1). Conversely, our approach based on T_1_‐weighting is sensitive to all vessels, which thus reflects total CBV and not only microvascular CBV, and can provide a more complete picture of the vascular response. Indeed, we found overlap of activation at the shortest TE with both macrovessels and the parenchyma (Figure 1).

### 4.5 Comparison to other UTE/ZTE studies

Several recent studies have explored the feasibility of minimal TE‐based fMRI for detecting functional responses in awake rodent models. For instance, Valjakka et al.^58^ demonstrated the application of ZTE‐fMRI in detecting whisker‐evoked responses in rodents, reporting ˜0.4% signal change at 5 Hz stimulation with an in‐plane resolution of 0.625 mm. Imamura et al.^59^ also reported ZTE‐fMRI signal changes of ˜0.05% at FA of 1° and ˜0.12% at FA = 5° in response to electrical stimulation in mice, further emphasizing the challenges in sensitivity associated with minimal TE imaging. Additionally, MacKinnon et al.^60^ introduced a CBV‐sensitive technique called Steady‐state On‐the‐Ramp Detection of INduction‐decay with Oversampling (SORDINO), reporting ˜0.3% signal change during electrical stimulation using a volume transmit‐and‐surface receive coil configuration, comparable to our experimental hardware.

In our study, we observed ˜0.3% signal change in the visual cortex using UTE‐fMRI in mice, aligning with the response magnitudes reported in the aforementioned studies despite differences in contrast mechanisms and stimulation protocols. These findings support the capability of UTE‐based approaches to capture localized functional responses at high spatial resolution. Moreover, in contrast to previous studies focusing predominantly on parenchymal responses, we also observed signal changes at the pial surface. This may reflect an added sensitivity of our contrast mechanism to superficial vascular dynamics, potentially offering an advantage in resolving surface‐level hemodynamic activity.

### 4.6 Limitations

This study has some limitations. As the contrast agent enhances CBV of all blood vessels, the differential contribution of artery and vein could not be distinguished. Despite the acquisition of high resolution, the fMRI analysis required spatial smoothing for improving the SNR. This would impact a layer‐dependent analysis would be performed. It may be worth noting that because the acquired fMRI data has a different image contrast and spatial resolution to that of the anatomical reference data, accurate alignment of the very small pial ROI is challenging, and so slight misalignments are still possible.

## 5 CONCLUSIONS

In this study, we investigated the feasibility of conducting fMRI experiments using a T_1_‐weighted contrast combing UTE imaging and iron oxide nanoparticles in mice. Our findings demonstrate that, when TEs are sufficiently short to minimize $T_{2}^{*}$ weighting, a bright‐blood signal following the administration of a contrast agent is observed, as well as a positive fMRI signal change with activation. At longer TEs, we observed a negative signal change across all ROIs, as shown in previous studies. The positive fMRI signal changes seen in the parenchyma and on the pial surface suggest an involvement of surface vessels during functional hyperemia. These positive functional signal changes are consistent with blood volume responses to stimulation; however, further studies are needed to disentangle veins from arteries as well as potential contributions from inflow effects.

## FUNDING INFORMATION

This work was supported by an Australian Government Research Training Program Scholarship, The National Health and Medical Research Council (NHMRC)–National Institutes of Health (NIH) BRAIN Initiative Collaborative Research Grant (APP1117020), and the NIH National Institute of Mental Health (NIMH) BRAIN Initiative Grant (R01‐MH111419).

## Data Availability

The data and code are available from the corresponding author upon reasonable request.
